# Editorial: The Expansion of Female Fertility

**DOI:** 10.3389/frph.2021.781019

**Published:** 2022-01-27

**Authors:** Anat Hershko Klement, Galia Oron, Yaakov Bentov

**Affiliations:** ^1^Faculty of Medicine, Hebrew University of Jerusalem, Jerusalem, Israel; ^2^Division of Obstetrics and Gynecology, Hadassah-Hebrew University Medical Center, Jerusalem, Israel; ^3^Department of Obstetrics and Gynecology, Hadassah Mount Scopus-Hebrew University Medical Center, Jerusalem, Israel; ^4^Department of Obstetrics and Gynecology, Carmel Medical Center, Haifa, Israel; ^5^Department of OBGYN, Faculty of Medicine Technion, Haifa, Israel

**Keywords:** *in vitro* fertilization, IVF, female fertility, diminished ovarian reserve, POI, hippo pathway, endometrial senescence

The modernization and availability of medical services in the past century led to an unprecedented increase in lifespan and a changed perception of the pace of life. Together with an increased participation of women in the labor force and academic life led to a progressive delay in childbirth. However, despite an almost doubling of the average lifespan for women, the past century did not change the length of the fertile lifespan for women to any degree. As a result many industrialized countries are facing a drastic decrease in fertility rates to levels too low to maintain the size of the population and prevent its aging (1). Advanced maternal age affects both the residual quantity of oocyte containing follicles as well as their quality. The lack of sufficient number of functional follicles is referred to as diminished ovarian reserve (DOR). The condition has been poorly defined due to a lack of a consensus definition or a reliable operator independent marker. Recently AMH has emerged as the most likely candidate to become a basis for the diagnosis of DOR (2). Also, the most recent consensus definition of DOR “POSEIDON” considers the combined quantitative (AMH) and qualitative decline by defining DOR is an incapacity to achieve an euploid embryo (3). The rate of loss of ovarian follicles may in some women occur even faster leading to premature ovarian insufficiency (POI). It is estimated that 1–2% of women will develop failure of ovarian function before the age of 40 (4). This general trend of delaying childbirth and especially patients with POI poses a challenge as attempts to conceive toward the end of reproductive life are hindered by a decline in ovarian reserve, oocyte quality and the percentage of euploid embryos thus leading to a poor live birth rate. While the solution of conception *via* oocyte donation had been in practice for over two decades and is well-established, researchers and clinicians have been struggling to develop strategies to improve the chances of women at this age group to give birth to a genetically linked child. Recent developments in the understanding of the process of ovarian senescence led the several potential interventions that may allow us to tackle these challenges. In this section of the journal we examined a few of these promising avenues (*The Expansion of Female Fertility*). In the paper written by Vo and Kawamura et al., the authors address an observation that about half of the women diagnosed POI still have dormant follicles in their ovaries, and that mechanical disruption of the ovarian cortex following incision, drilling or fragmentation may result in the recruitment and development of these follicles Vo and Kawamura. Study of the phenomenon led to the establishment of the Hippo pathway's role in regulating the rate of recruitment of ovarian follicles. It was shown that inhibition of the hippo pathway leads to recruitment of follicles and that it could be induced by means of mechanical fragmentation or *via* activation of the AKT/FOXO3 pathway. The first is mediated *via* polymerization of the actin protein into filaments which in turn leads to the nuclear translocation of the Yes-associated protein (YAP). The increased nuclear concentration of YAP in turn leads to increased expression of a CCN growth factor and the BIRC apoptosis inhibitor. Similar effect may be achieved *via* activation of the AKT/FOXO3 pathway that promotes primordial follicle activation, by either Kit ligand or PTEN inhibition. The authors describe a combined approach involving both ovarian cortex fragmentation and AKT stimulation to women with POI and histologic evidence of dormant follicles. This tissue was then autografted under the tubal serosa and treated with gonadotropins and *in vitro* fertilization. This strategy' named *in vitro* Activation (IVA) was associated with over 40% response as well as the formation of clinical pregnancies and anecdotal live births. IVA had also been tested in women with diminished ovarian reserve secondary to advanced maternal age, showing improved oocyte yield, fertilization rate and embryo quality. This promising technology is still at its initial phases. It needs to be validated in larger longitudinal studies to assess its efficacy over time.

Another approach to is to optimize IVF treatment protocols in this challenging group of patients having a low ovarian reserve. It is well-established that increasing the daily dose of gonadotropins over 300 IU is ineffective (5). However, the jury is still out on whether a milder stimulation may be as effective. The paper by Le et al. describes a retrospective study that compared a large cohort of female fertility patients that were classified as POSEIDON group 4 (35 years or older with low AMH and AFC). These patients were allocated to be treated either with a standard high gonadotropin antagonist protocol or a mild protocol that included a sequential treatment with clomiphene citrate followed by low dose menotropin. Although the group of patients allocated to the mild stimulation protocol had a significantly lower ovarian reserve, the ongoing pregnancy rate between the two groups was not different. A similar finding was reported by Pilehvari et al., Ragni et al., Revelli et al., and Youssef et al. and warrants further validation with a large, randomized control trial (6–9).

Senescence is not limited to ovarian function. Recent publications implicated the presence of senescent decidual cells in the pathophisiology of implantation failure (10). The study by Song et al. included in this section provides a comparison between two interventions for resistant endometrium. The authors analyse the response to G-CSF with or without transcutaneous electrical acupoint stimulation (TEAS). The authors show an equally favorable endometrial thickness in both groups but a superior implantation rate in those treated with both G-CSF and TEAS.

Our improved understanding of aging in general and specifically of the reproductive system may potentially close the gap between the expansion of longevity and fecundity.

## Author Contributions

All authors listed have made a substantial, direct, and intellectual contribution to the work and approved it for publication.

## Conflict of Interest

The authors declare that the research was conducted in the absence of any commercial or financial relationships that could be construed as a potential conflict of interest.

## Publisher's Note

All claims expressed in this article are solely those of the authors and do not necessarily represent those of their affiliated organizations, or those of the publisher, the editors and the reviewers. Any product that may be evaluated in this article, or claim that may be made by its manufacturer, is not guaranteed or endorsed by the publisher.
